# CauReL: Dynamic Counterfactual Learning for Precision Drug Repurposing in Alzheimer’s Disease

**DOI:** 10.21203/rs.3.rs-8206648/v1

**Published:** 2025-12-15

**Authors:** Yanfei Wang, Minghao Zhou, Zijia Tang, Chenxi Xiong, Breton Asken, Baijian Yang, Jing Su, Xiaobo Zhou, Qianqian Song

**Affiliations:** 1 Department of Health Outcomes and Biomedical Informatics, College of Medicine, University of Florida, Gainesville, FL, USA; 2 Trinity College, Duke University, Durham, NC, USA; 3 School of Applied and Creative Computing, Purdue University, West Lafayette, IN, USA; 4 Department of Clinical and Health Psychology, University of Florida, Gainesville, FL, USA; 5 Department of Biostatistics and Health Data Science, Indiana University School of Medicine, Indianapolis, IN, USA; 6 Center for Computational Systems Medicine, McWilliams School of Biomedical Informatics, The University of Texas Health Science Center at Houston, Houston, TX, USA

**Keywords:** Causal AI, Counterfactual representation learning, Individualized Treatment Effects (ITE), Drug Repurposing, Electronic Health Records (EHRs), Alzheimer’s Disease (AD)

## Abstract

Alzheimer’s disease has few effective therapies, and decades of amyloid- and tau-focused trials have delivered only modest benefit with substantial toxicity. Drug repurposing using real-world data offers a faster and lower-risk route to new treatments, yet current approaches typically average effects across populations, model disease onset and progression separately, and provide little insight into which patients are most likely to benefit. We present CauReL, a dynamic counterfactual representation learning framework that enables transparent, patient specific estimation of treatment effects from large-scale electronic health records for precision drug repurposing in AD. CauReL first learns balanced latent representations of treated and untreated patients using Integral Probability Metric regularization, then jointly predicts two clinically linked outcomes, incident AD and time from mild cognitive impairment (MCI) to AD, to generate paired counterfactual outcomes for every individual. A counterfactual explanation module quantifies how clinical features shape benefit at the patient level, and uplift trees transform complex heterogeneity into simple, rule-based subgroups suitable for trial enrichment and clinical decision support.

Using independent cohorts from OneFlorida+ and All of Us, we screened outpatient prescriptions with at least 20 percent exposure among 28,605 individuals with mild cognitive impairment, of whom 4,990 progressed to Alzheimer’s disease. CauReL substantially improved covariate balance and distributional overlap across drug cohorts and achieved strong predictive accuracy for both incidence (AUC greater than 0.90) and progression timing (C index 0.81 to 0.84; Spearman 0.80 to 0.86). Twenty drugs showed consistent protective associations, with four emerging as highly reproducible across both networks, the metabolic agents liraglutide and empagliflozin and the neuroactive agents entacapone and amantadine. These drugs were associated with meaningful absolute risk reductions and clinically significant delays in progression from mild cognitive impairment to Alzheimer’s disease. Metabolic drugs produced the strongest benefits in individuals with diabetes, obesity, or cardiovascular disease, whereas neuroactive drugs provided broadly consistent protection across most subgroups.

CauReL is available as an open source Python package with a companion web server for direct application to new cohorts or disease settings (https://caurel.site/). This work delivers a scalable and interpretable framework for prioritizing repurposable drugs and designing targeted clinical trials for the patients most likely to benefit.

## INTRODUCTION

Among the various forms of dementia, Alzheimer’s disease (AD) is the most prevalent subtype, accounting for 60–80% of all cases worldwide^1^. In the United States, AD is the sixth leading cause of death^2^, affecting over 6 million Americans today, and this number is projected to more than double to 13.8 million by 2060^3^. In addition, many millions more are living with mild cognitive impairment (MCI), a prodromal stage of AD, placing them at high risk for disease progression^4^. The socioeconomic burden is substantial, the annual cost of care for AD and related dementias is expected to exceed US $1 trillion by 2050^2^, encompassing direct medical care, long-term institutional care, informal caregiving, and the loss of productivity and quality of life. Despite sustained research efforts and significant funding, effective therapeutics remain scarce. Over the last twenty years, numerous trials^5,6^ targeting pathological hallmarks^7^, amyloid-β aggregation^8^, tau pathology^9^, neuroinflammation^10^, oxidative stress^11^, have not demonstrated significant clinical benefit, or have yielded only modest gains accompanied by notable adverse effects. Even recently approved drugs, such as Lecanemab for early AD or MCI with amyloid pathology, have shown only a 27–30% slowing of cognitive decline over 18 months versus placebo^12^, while raising concerns about amyloid-related imaging abnormalities and edema. Similarly, drugs like Crenezumab failed to meet primary endpoints in phase III trials, despite indications of potential benefit in specific subgroups^13^.

Drug repurposing, the identification of new therapeutic uses for existing drugs, offers a pragmatic and cost-effective strategy to accelerate therapeutic discovery in AD^14^. Repurposed drugs benefit from prior knowledge of safety and pharmacokinetics, shortening the path to clinical testing^15^. The expanding availability of real-world data, particularly large electronic health records (EHRs), enables longitudinal target-trial emulations in diverse populations, allowing systematic comparisons of drug exposures and outcomes and prioritization of candidates for prospective study. To convert these comparisons into credible evidence of benefit, causal inference is required, with rigorous control of confounding. Inverse probability of treatment weighting (IPTW) based on propensity scores is a common approach for covariate balance in treated and untreated groups and is widely used in target-trial emulations and high-throughput repurposing pipelines^16–18^. For instance, one high-throughput target trial emulation leveraged IPTW to explore potential drug candidates for AD^19^. Deep learning frameworks have also been adapted to drug repurposing, with approaches such as long short-term memory networks combined with IPTW to estimate treatment effects, thereby enhancing precision in effect estimation^20^.

Despite these advances, critical methodological gaps remain. First, IPTW-based pipelines estimate average treatment effects (ATEs) and assume that all patients respond similarly^21–23^. This assumption conflicts with the substantial clinical heterogeneity of AD^24–28^, where comorbidities and baseline characteristics can produce markedly different responses. As a result, a drug with a neutral population level effect may still provide meaningful benefit to well defined subgroups, but these benefits are often obscured when analyses rely solely on ATEs. Second, population level ATEs do not yield patient specific risk benefit estimates and therefore cannot guide treatment selection for a new individual. Clinicians require individualized predictions rather than a single cohort wide average to decide whether a repurposed drug is appropriate for a particular patient. Third, most current AD repurposing pipelines model disease incidence and progression in separate analyses, despite the fact that an effective therapy should ideally reduce the likelihood of developing AD and slow conversion from mild cognitive impairment. Treating these endpoints independently can lead to inconsistent effect estimates and conflicting subgroup priorities. Together, these limitations constrain the ability of existing methods to support precision drug repurposing in AD.

Here, we introduce CauReL, a dynamic counterfactual representation learning framework that directly addresses these limitations and enables precision drug repurposing in AD. First, CauReL moves beyond population level ATEs by learning balanced latent representations of treated and untreated patients and estimating paired potential outcomes for each individual, which exposes heterogeneous benefits that conventional IPTW based analyses obscure. Second, the framework provides individualized risk and benefit predictions for both AD onset and progression, allowing treatment effects to be applied to new patients and supporting subgroup specific decision making rather than cohort wide averaging. Third, CauReL models these two clinically linked endpoints within a unified architecture, ensuring internally consistent inferences about incident disease and disease trajectory, which current pipelines often treat separately. A counterfactual explanation module attributes treatment benefit to specific clinical features, and a white box estimator summarizes heterogeneous effects into transparent subgroup rules suitable for trial enrichment. Applied to OneFlorida+ and All of Us, CauReL reveals reproducible patient level heterogeneity and identifies drug candidates that are both protective and generalizable, offering a rigorous and interpretable framework for precision drug repurposing. An accompanying web server is available at https://caurel.site/ for direct application.

## RESULTS

### Overview of population cohorts and CauReL architecture

**Figure 1.**
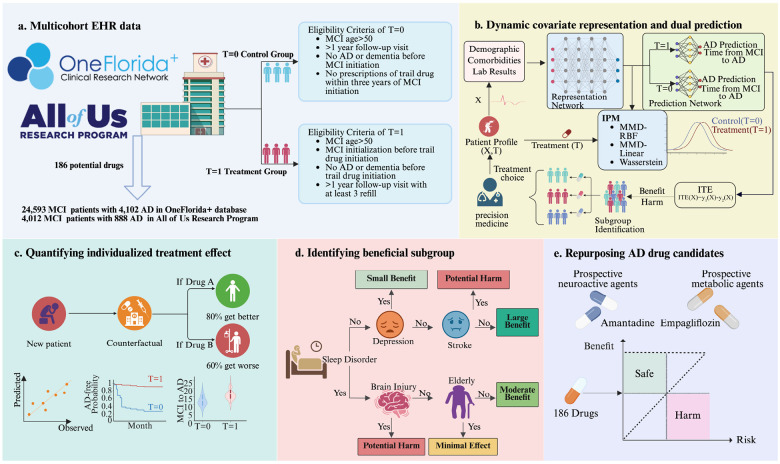
Study Cohort Assembly Flowchart. **(a)** Study design and population. Electronic health record (EHR) data from the OneFlorida+ network (24,593 MCI patients, 4,102 AD conversions) and the All of Us program (4,012 MCI patients, 888 AD conversions) were analyzed. Drugs prescribed to at least 20 % of MCI patients were retained, yielding 186 candidates. Treated patients (T = 1) initiated the candidate drug after MCI diagnosis and maintained ≥ 3 refills during ≥ 1 year of follow-up; controls (T = 0) had no exposure within 5 years of MCI diagnosis. **(b)** Model architecture. CauReL combines a representation network that balances treated and untreated groups via Integral Probability Metric (IPM) regularization with a dual-prediction network that models AD onset and progression time under both treatment and control. **(c)** Quantifying treatment effects. The framework estimates individualized treatment effects (ITEs) aligned with observed outcomes. **(d)** Interpretable subgroup discovery. CFR-based uplift trees reveal rule-based subgroups associated with benefit or harm. **(e)** Clinical translation. Benefit-risk mapping identifies four reproducible for AD repurposing.

To evaluate the CauReL’s findings for AD drug repurposing, we assembled independent discovery and validation cohorts from two large EHR networks. The discovery cohort was drawn from the OneFlorida+ Clinical Research Network, which includes longitudinal records for more than 15 million individuals in Florida and neighboring states. The validation cohort was drawn from the All of Us Research Program. To ensure comparability, we harmonized baseline covariates across cohorts, including demographics, comorbidities, laboratory measurements, and medication histories. We screened outpatient prescriptions of OneFlorida+ and restricted analyses to drugs with at least 20 percent exposure among patients with MCI to ensure adequate power, yielding 186 candidates spanning metabolic, cardiovascular, neuroactive, and anti-inflammatory classes. For each drug, treated and comparison groups were defined from medication histories, see Fig. 1a. Treated patients, T=1, were age 50 or older, had MCI before first exposure, no prior AD or other dementia, at least one year of follow up, and adherence defined as three or more refills. Comparison patients, T=0, met the same age and follow up criteria, had no prior AD or dementia, and no prescriptions for the same drug within three years of MCI onset. In OneFlorida+, we identified 24,593 patients with MCI, including 4,102 who converted to AD within one year. In All of Us, we identified 4,012 patients with MCI, including 888 who progressed to AD during follow up.

CauReL integrates representation learning with counterfactual modeling to estimate ITE for AD onset and progression (Fig. 1b). The framework first learns balanced latent representations of treated and untreated patients using Integral Probability Metric regularization, implemented through MMD Linear, MMD RBF, and Wasserstein distance, to reduce confounding and improve comparability. On this balanced representation, a dual prediction network outputs the paired potential outcomes for each patient, under treatment and under no treatment, for two linked endpoints: probability of AD onset and months from MCI to AD. These paired outputs constitute the counterfactual predictions and allow CauReL to compute patient specific ITEs that quantify how a drug would change both AD risk and progression time.

Applications are shown in Fig. 1c–e. Counterfactual predictions closely match observed outcomes, supporting accurate estimation of individualized treatment effects rather than mere disease prediction (Fig. 1c). Uplift trees transform these counterfactual ITEs into simple, interpretable subgroup rules that highlight combinations such as depression, sleep disorder, traumatic brain injury, stroke, and age that separate high benefit from minimal benefit or potential harm (Fig. 1d). A benefit risk map then integrates these estimates across all screened drugs, positioning amantadine, entacapone, liraglutide, and empagliflozin in the high benefit, low risk quadrant and prioritizing them as leading candidates for AD repurposing (Fig. 1e).

### CauReL improves covariate balance and yields accurate counterfactual estimates for AD incidence

**Figure 2.**
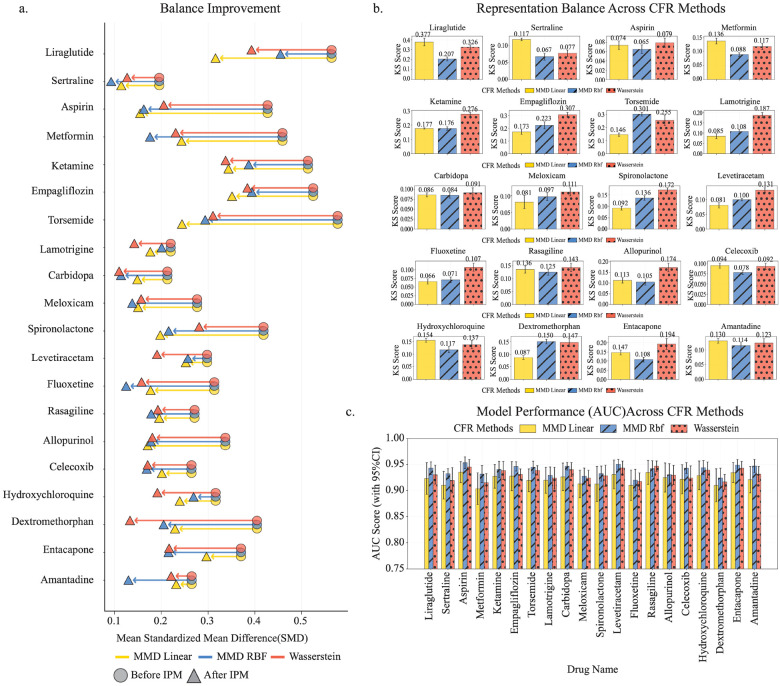
Evaluation of covariate balancing and predictive performance across IPM regularization strategies. **(a)** shows the standardized mean differences (SMDs) for baseline covariates between treated and control groups before and after representation learning using three Integral Probability Metric (IPM) configurations: MMD-Linear (yellow), MMD-RBF (blue), and Wasserstein distance (red). The x-axis represents individual drug candidates, and the y-axis represents the average SMD across all baseline covariates. Each drug is displayed as a line segment, where the circle denotes the pre-regularization SMD and the triangle denotes the post-regularization SMD. Longer line lengths indicate greater improvement in covariate balance, while higher starting SMDs reflect more substantial initial imbalance between treated and control groups. **(b)** presents the Kolmogorov-Smirnov (KS) distance between treated and control group distributions in the learned representation space. The x-axis lists individual drugs, while the y-axis shows the mean KS distance. Lower KS values indicate better alignment of the two distributions. Bars represent the mean KS value across 10-fold cross-validation, and error bars denote the standard deviation, reflecting the stability of each method. **(c)** displays the predictive performance of the counterfactual outcome model for identifying which MCI patients progress to AD, measured by the area under the receiver operating characteristic curve (AUC). The x-axis represents individual drug, and the y-axis represents the AUC value. All three IPM configurations achieved AUC values above 0.90, indicating strong predictive performance.

We systematically screened 186 drugs and identified 20 candidates with consistent and strong protective associations against AD across OneFlorida+ and All of Us. CauReL was rigorously evaluated for its ability to achieve covariate balance and enable accurate counterfactual prediction of AD progression from MCI. To assess covariate balance, we compared standardized mean differences (SMDs) in baseline covariates between treated and control groups, before and after our representation learning of CauReL (Fig. 2a). Using the three IPM regularization methods implemented in CauReL (MMD-Linear, MMD-RBF, and Wasserstein distance), we observed substantial improvements across all drugs. Prior to adjustment, most drugs showed substantial baseline imbalance, with initial SMDs ranging from 0.26 to 0.52. After adjustment, all three methods produced marked reductions in imbalance across nearly all drugs. For example, liraglutide’s SMD decreased from 0.49 to 0.32 using MMD-Linear, while empagliflozin declined from 0.50 to 0.34 under the same method. Amantadine achieved the lowest residual imbalance, dropping from 0.26 to 0.12 with MMD-RBF. Entacapone benefitted most from the Wasserstein approach, with its SMD reduced from 0.52 to 0.21. Across drugs, CauReL demonstrates superior capability to align treated and control populations, with MMD-RBF and Wasserstein distance showing greater ability than MMD-Linear.

We next quantified distributional overlap between treated and control groups in the learned representation space using the Kolmogorov-Smirnov (KS) distance (Fig. 2b). Lower KS values indicate improved balance. CauReL, particularly with MMD-RBF, achieved consistent reductions in KS distance across drugs, with variability assessed via 10-fold cross-validation. For example, liraglutide achieved a KS distance of 0.207 with MMD-RBF, compared to 0.377 with MMD-Linear and 0.326 with Wasserstein. Empagliflozin showed the lowest divergence under MMD-Linear (0.173) versus 0.223 (MMD-RBF) and 0.307 (Wasserstein). For entacapone, MMD-RBF achieved the smallest KS distance (0.108) compared to 0.147 (MMD-Linear) and 0.194 (Wasserstein). Similarly, amantadine achieved 0.114 under MMD-RBF, compared to 0.130 (MMD-Linear) and 0.127 (Wasserstein). Finally, we evaluated the accuracy of CauReL’s counterfactual outcome predictions for AD onset, which form the basis of ITE (Fig. 2c). Predictive performance, measured by the area under the receiver operating characteristic curve (AUC), confirmed that strong discriminative ability across all configurations (AUC > 0.90). Among the three IPM configurations, MMD-RBF consistently delivered the highest performance, with mean AUCs exceeding 0.93 across multiple drugs, while MMD-Linear and Wasserstein achieved slightly lower yet comparable accuracy.

### CauReL identifies candidate drugs with reproducible protective effects across OneFlorida+ and All of Us

**Figure 3.**
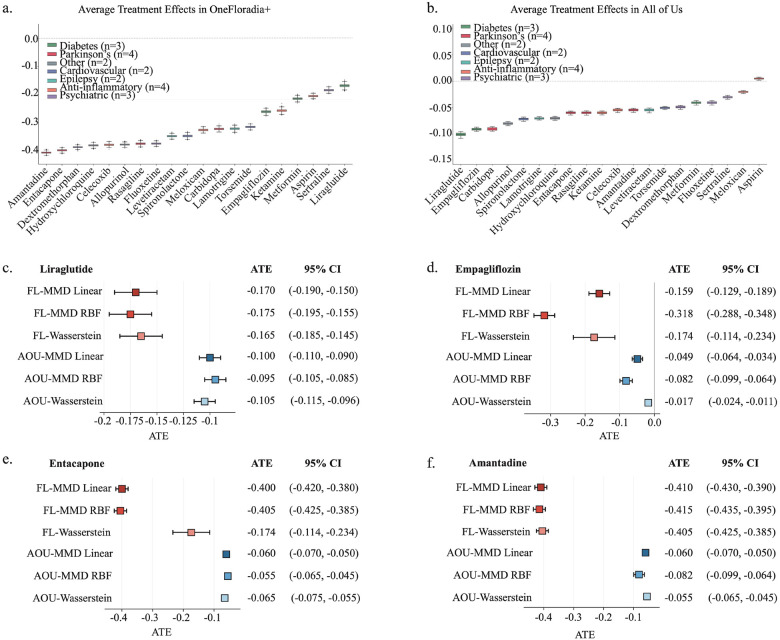
Identification of drug candidates and cross-cohort validation of treatment effects. **(a)** and **(b)** show the ranked average treatment effects (ATEs) for progression from MCI to AD across 20 drug candidates. The x-axis lists the drugs in order of effect size, while the y-axis shows the estimated ATE, where more negative values indicate stronger protective associations. **(a)** shows results from the OneFlorida+ discovery cohort, and **(b)** shows results from the All of Us validation cohort. **(c-f)** provide ATEs and 95% confidence intervals (CIs) of the four top candidate drugs: liraglutide **(c)**, empagliflozin **(d)**, entacapone **(e)**, and amantadine **(f)**. FL- and AOU- prefixes indicate estimates from the OneFlorida+ and All of Us datasets, respectively. Bar colors represent the regularization method: yellow for MMD-Linear, blue for MMD-RBF, and red for Wasserstein distance. Negative ATEs indicate that patients receiving the drug were less likely to progress from MCI to AD compared with untreated individuals.

The estimated ATEs on AD incidence across 20 candidate drugs are ranked from most to least protective in Figure 3a (OneFlorida+) and Figure 3b (All of Us). A more negative ATE indicates a stronger protective effect, corresponding to a greater reduction in the risk of progression from MCI to AD among treated patients. Four top-ranking drugs were prioritized for detailed evaluation, two metabolic drugs from All of Us (liraglutide and empagliflozin) and two neuroactive drugs from OneFlorida+ (entacapone and amantadine).

Liraglutide, a glucagon-like peptide-1 (GLP-1) receptor agonist used for type 2 diabetes and obesity^29^, demonstrated a consistent and robust protective effect across both cohorts (Fig. 3c). In All of Us, CauReL with MMD-RBF estimated an ATE of −0.095 (95% CI: −0.105 to −0.085), while in OneFlorida+, the effect reached approximately −0.17 (e.g., CauReL with MMD-RBF: −0.175, 95% CI: −0.195 to −0.155). Liraglutide’s improvements in insulin sensitivity, vascular integrity, and neuroinflammatory regulation may contribute to its observed neuroprotective effect. The consistent direction and magnitude across datasets position liraglutide as a compelling candidate for AD risk reduction. Empagliflozin, a sodium-glucose cotransporter-2 (SGLT2) inhibitor used for diabetes and heart failure^30–32^, produced comparable protective effects (Fig. 3d). In All of Us, CauReL with MMD-Linear estimated an ATE of −0.049 (95% CI: −0.064 to −0.034), while in OneFlorida+, the protective effect was larger (−0.159, 95% CI: −0.189 to −0.129). These consistent negative estimates suggest that empagliflozin may confer cognitive benefits through improved glycemic control, vascular protection, and systemic anti-inflammatory pathways.

Entacapone, a catechol-O-methyltransferase (COMT) inhibitor approved for Parkinson’s disease^33,34^, exhibited one of the strongest protective effects in OneFlorida+ (Fig. 3e), with ATEs ranging from −0.38 to −0.40. Although the magnitude was smaller in All of Us (e.g., CauReL with MMD-RBF: −0.055, 95% CI: −0.065 to −0.045), the uniformly negative direction across datasets supports the reproducibility of its estimated treatment effect and suggests a dopaminergic contribution to neuroprotection. Finally, amantadine, an N-methyl-D-aspartate (NMDA) receptor antagonist with dopaminergic and antiviral properties^35^, produced the largest protective effect among all evaluated drugs (Fig. 3f). In OneFlorida+, estimated ATEs ranged from −0.405 to −0.415 with narrow confidence intervals (e.g., CauReL with MMD-RBF: −0.415, 95% CI: −0.435 to −0.395). Although the effect size was smaller in All of Us (e.g., −0.082, 95% CI: −0.099 to −0.064), the consistent negative direction underscores amantadine’s strong potential for delaying cognitive decline.

### CauReL-derived ITEs reveal subgroup-specific and comorbidity-dependent drug responses

**Figure 4.**
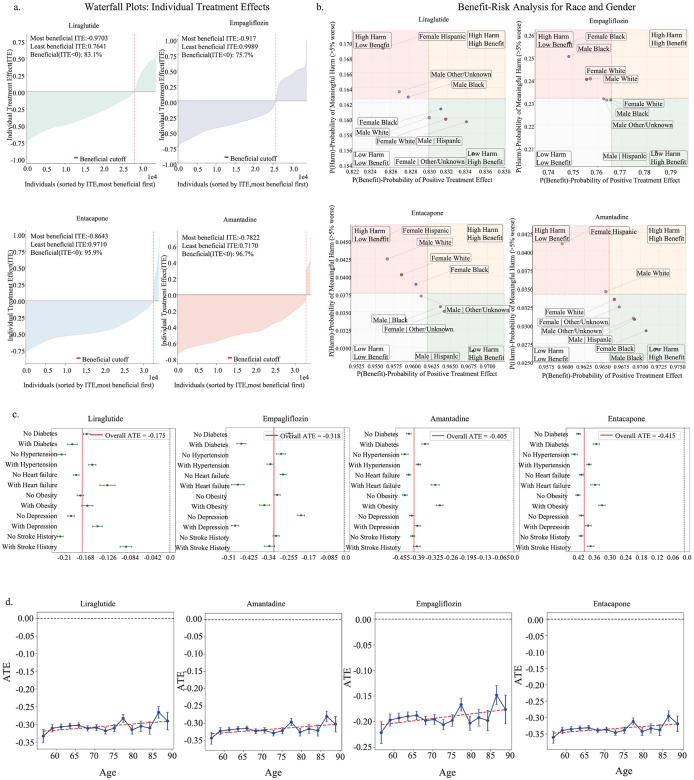
Subgroup analyses reveal heterogeneity of treatment effects **(a)** presents waterfall plots of individualized treatment effects (ITEs) for each drug, sorted from most to least beneficial. The x-axis represents individual patients, while the y-axis shows the ITE value. Negative ITEs indicate a protective effect against progression to AD, with the proportion of negative values reflecting the consistency of benefit across patients. **(b)** visualizes subgroup-level heterogeneity stratified by race and sex. The x-axis represents the probability that individuals within a subgroup experience a beneficial treatment effect, while the y-axis represents the probability of high variability or inconsistent effects within that subgroup. Subgroups in the lower-right quadrant exhibit both high benefit and low variability, indicating the most reliable and clinically promising populations. **(c)** shows ATEs stratified by comorbidity status, including diabetes, hypertension, heart failure, obesity, depression, and stroke. The x-axis lists comorbidities, while the y-axis shows the ATE, with darker bars indicating patients with the comorbidity and lighter bars indicating patients without it. Negative values represent protective associations. **(d)** depicts the mean ATEs by age group, where the x-axis represents age categories (50–59, 60–69, 70–79, ≥80), and the y-axis represents the mean ATE. Error bars indicate variability within each age group.

To further characterize heterogeneity in treatment response, we conducted subgroup analyses for the top drug candidates based on ITE estimates. These subgroup analyses provided a detailed view of how the magnitude of benefit varied across patients. Fig. 4a presents waterfall plots of ITEs for each drug, ordered from the most to the least beneficial individuals. Most patients showed negative ITEs, indicating protection against MCI-to-AD progression. Metabolic drugs, liraglutide and empagliflozin, showed wider spread, with a small subgroup near neutral effect. Neuroactive drugs, entacapone and amantadine, were highly consistent, with benefit in more than 95 percent of individuals. Fig. 4b summarized the probability of achieving a meaningful protective effect across race and sex. Subgroups in the lower right quadrant had high benefit probability and low variability, those in the upper left had weaker and less consistent effects. For liraglutide, most subgroups were favorable, with smaller and less consistent effects in female Hispanic and male Black groups, and stronger profiles in female Black, female White, and male Hispanic groups. Empagliflozin showed greater dispersion. Black men and women, and White men and women, had lower benefit probabilities, while male Hispanic participants showed distinct and consistent benefit. Entacapone responses were more balanced overall, with the strongest profiles in male Black and male Hispanic groups. Amantadine showed broadly favorable profiles, with high benefit in female White, female Black, and male Black groups, and comparatively weaker effects in female Hispanic and male White groups. **Supplementary Fig. 1a** in All of Us showed the same directional trends, with lower harm probabilities and tighter clustering.

To evaluate how baseline health conditions influence drug efficacy, Fig. 4c compares ATEs between patients with and without common comorbidities, including diabetes, hypertension, heart failure, obesity, depression, and stroke history. Effects remained protective across drugs, but magnitudes varied by health profile. Liraglutide and empagliflozin showed the largest benefits in diabetes, with additional gains in obesity, hypertension, or heart failure, consistent with cardiometabolic mechanisms^36,37^. Amantadine and entacapone showed stronger protection in patients without these comorbidities. This pattern is consistent with their predominantly central mechanisms, amantadine acting through dopaminergic and glutamatergic modulation^38^ and entacapone through catechol-O-methyltransferase (COMT) mediated enhancement of dopaminergic signaling^39^, which primarily affect neural circuits rather than systemic metabolic risk. Comorbidity-stratified results replicated in All of Us, confirming stability across settings, see **Supplementary Fig. 1b**. Age-stratified ATEs remained negative across late adulthood, with gradual attenuation at older ages and no direction reversal (Fig. 4d). All of Us showed the same pattern, see **Supplementary Fig. 2a**.

### Counterfactual interpretability maps distinct feature driven response profiles for metabolic and neuroactive drugs

**Figure 5.**
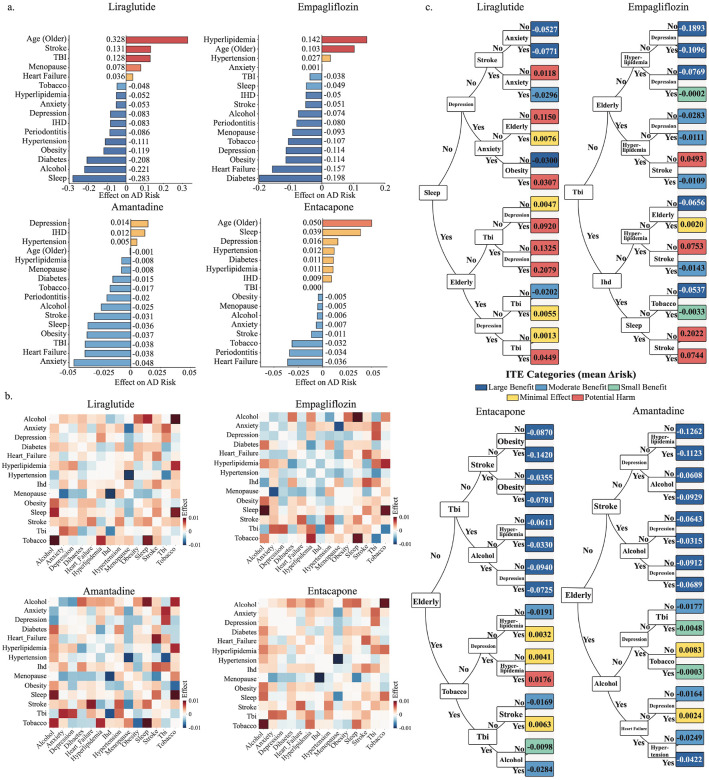
Feature importance and interaction toward AD **(a)** shows directional feature importance, where the x-axis lists baseline features and the y-axis shows their directional impact on predicted AD risk. Features with bars pointing to the left are associated with enhanced drug protection, while those pointing to the right are linked to attenuated drug effects. **(b)** provides a heatmap of pairwise interaction effects between clinical and demographic features. The x- and y-axes represent individual features. Red cells indicate synergistic effects, where the combined risk is greater than additive, while blue cells indicate antagonistic or overlapping effects. Cells outlined in black represent clinically relevant interactions. **(c)** displays uplift decision trees that segment patients into groups with varying levels of benefit. Deep blue nodes represent subgroups with the largest predicted benefit, light blue nodes represent moderate benefit, yellow nodes indicate minimal benefit, and red nodes indicate potential harm.

We quantified directional feature effects by setting one binary covariate to present and to absent in turn, holding all other covariates fixed, and measuring the change in the treated potential outcome (Fig. 5a), Rightward effects indicate higher treated risk, leftward effects indicate greater protection. Metabolic and neuroactive drugs showed a clear split. For liraglutide, older age, stroke, and traumatic brain injury increased treated risk, whereas diabetes, obesity, alcohol misuse, and sleep disorder reduced treated risk, indicating stronger benefit consistent with neurovascular and neuroendocrine modulation. Empagliflozin displayed a similar cardiometabolic profile, with hyperlipidemia, hypertension, and older age increasing treated risk and diabetes, obesity, and heart failure associated with risk reduction, consistent with cardio-renal-metabolic efficacy. In contrast, amantadine and entacapone were broadly comorbidity insensitive. Amantadine showed small risk reductions across features, and entacapone showed similar stability with added benefit in heart failure, periodontitis, and stroke, suggesting modulation of neurovascular inflammation and oxidative stress. Patterns replicated in All of Us, supporting cross-cohort robustness (**Supplementary Fig. 2b**).

To further dissect how combinations of patient factors shape treatment response, we examined pairwise feature interactions (Fig. 5b). Across all four drug cohorts, several clinically coherent and mechanistically plausible risk interaction patterns emerged. Traumatic brain injury (TBI) exhibited the strongest and most consistent risk amplification when co-occurring with anxiety or depression, underscoring the compounded vulnerability of patients with neuropsychiatric sequelae of trauma. This observation aligns with the established bidirectional relationship between neural injury, affective dysregulation, and neurodegeneration^40^. Lifestyle-related exposures, such as tobacco and alcohol use, also frequently amplified risk, supporting evidence that vascular inflammation and circadian disruption jointly accelerate AD progression^41^. A particularly distinctive pattern involved the combination of alcohol abuse with anxiety or depression. For liraglutide and amantadine, this combination was associated with risk amplification (red regions), indicating that patients with neurobehavioral stressors remained more susceptible to AD despite treatment. In contrast, empagliflozin and entacapone showed risk attenuation (blue regions) under the same conditions, suggesting preserved protection even among high-risk individuals.

Building on the interaction patterns identified above, Fig. 5c presents the CFR-based uplift trees, which convert CauReL’s counterfactual estimations into transparent, rule-based representations of treatment response. Each tree summarizes how combinations of patient factors stratify individuals into subgroups with distinct levels of benefit or potential harm, revealing interpretable decision paths that explain who benefits most and under what conditions. For liraglutide, sleep disorder was the top splitter, with maximal benefit in patients without stroke or depression and reduced benefit in those with TBI plus depression. For empagliflozin, absence of TBI and younger age predicted the largest risk reduction, while hyperlipidemia without stroke shifted toward potential harm. For amantadine, age was dominant, with the greatest benefit in younger, stroke-free, depression-free, normolipidemic patients and minimal benefit in older patients with depression or heart failure. Entacapone showed a similar age-anchored structure, with best outcomes in stroke-free, non-obese, non-hyperlipidemic patients and diminished benefit when hyperlipidemia co-occurred.

### Identified repurposing candidates demonstrate delayed progression from MCI to AD

**Figure 6.**
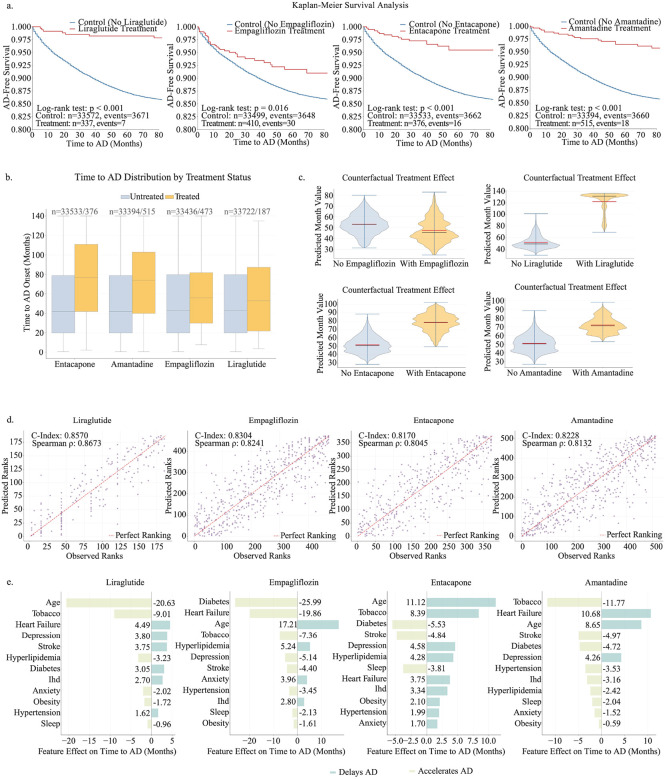
Effects of top candidate drugs on delaying AD progression **(a)** presents Kaplan-Meier survival curves comparing treated patients and matched controls for time to conversion from MCI to AD. The x-axis represents months since MCI diagnosis, and the y-axis represents the probability of remaining free from AD. Red curves represent treated patients, and blue curves represent matched controls. Clear separation between curves indicates slower progression among treated patients, with all log-rank tests yielding p < 0.05. **(b)** summarizes the original observed outcomes, showing the distribution of times to AD conversion for treated (red) versus untreated (blue) patients. The x-axis lists the four candidate drugs, and the y-axis represents time in months. **(c)** shows the counterfactual model predictions for each drug. Violin plots depict the predicted distribution of conversion times under two scenarios: no treatment (blue, left) and treatment (red, right). Wider sections indicate higher densities of patients at a given predicted conversion time, and vertical black bars mark the median prediction. **(d)** compares predicted versus observed treatment effects for delaying AD conversion. The x-axis represents the model-predicted delay, and the y-axis represents the observed delay. Each point represents a patient, with concordance index (C-index) values between 0.81 and 0.84 and Spearman correlations between 0.80 and 0.86, demonstrating strong agreement between predicted and actual effects. **(e)** illustrates the directional feature importance for each drug’s counterfactual model. The x-axis represents the magnitude of the feature’s influence on predicted time to AD conversion, while the y-axis lists key clinical and demographic features (e.g., age, comorbidities). Red bars indicate features associated with increased AD risk (shorter time to conversion), whereas green bars indicate protective features associated with delayed progression. Each subpanel corresponds to one of the four candidate drugs: liraglutide, empagliflozin, entacapone, and amantadine.

To assess whether the identified drugs not only reduced AD risk but also slowed disease progression, we analyzed time from MCI to AD as a continuous outcome. As shown in Fig. 6a, Kaplan Meier curves showed clear separation between treated (red) and matched control (blue) groups for all four candidates, with treated patients consistently exhibiting longer AD free survival in both the OneFlorida+ discovery cohort and the All of Us validation cohort (all log rank p < 0.05). The reproducibility of these patterns in All of Us (**Supplementary Fig. 3a**) supports the robustness and generalizability of CauReL derived effects across populations. Observed conversion times further supported these protective associations (Fig. 6b), with median delays of approximately 5.4 months for liraglutide and 4.8 months for empagliflozin, and consistent but smaller delays of about 3 to 4 months for amantadine and entacapone.

Counterfactual validation from the OneFlorida+ cohort (Fig. 6c) demonstrated consistent rightward and upward shifts in the model-predicted time to AD conversion under treated (red) versus untreated (blue) scenarios, indicating delayed disease onset across all four drugs. The mean model-predicted times to AD onset were 28.1 ± 0.4 months for amantadine, 24.3 ± 0.5 months for liraglutide, 18.1 ± 0.6 months for empagliflozin, and 32.2 ± 0.5 months for entacapone, compared with corresponding control means of 25.3 ± 0.4, 21.5 ± 0.4, 15.4 ± 0.5, and 28.2 ± 0.4 months, respectively. The estimated average treatment effects (ATEs) were +2.8 months for amantadine (95% CI, +2.0 to +3.7), +4.1 months for liraglutide (95% CI, +3.2 to +4.9), +2.7 months for empagliflozin (95% CI, +1.9 to +3.4), and +4.0 months for entacapone (95% CI, +3.1 to +4.8). The validation in the All of Us cohort (**Supplementary Fig. 3b**) reproduced nearly identical trends, with treated patients exhibiting right-shifted distributions and comparable mean delays: +2.6 months for amantadine, +3.8 months for liraglutide, +2.5 months for empagliflozin, and +3.9 months for entacapone. This predictive accuracy and consistency are summarized in Fig. 6d, where the model achieved strong agreement between predicted and observed progression times (C-index = 0.81–0.84; Spearman r = 0.80–0.86), validating its capacity to rank patient-level risk and treatment response. Finally, Fig. 6e highlights the directional feature importance underlying these effects. For metabolic drugs, comorbid diabetes, obesity, and hypertension were the strongest predictors of delayed progression, consistent with their roles in glycemic and vascular regulation. In contrast, the neuroactive drugs displayed broadly uniform benefits, with age (+0.05 effect) and stroke history (+0.03) emerging as mild risk enhancers rather than dominant modifiers.

## DISCUSSION

In this study, we developed and validated our CauReL framework to advance precision drug repurposing for AD. Unlike traditional approaches based on IPTW or other ATEs, our method directly estimates ITEs, allowing us to detect subgroup-specific differences in drug response that are often hidden in population-level analyses. The framework integrates two core components: a representation network that reduces confounding by aligning treated and untreated groups, and a dual prediction network that simultaneously models two key clinical outcomes: whether a patient develops AD and the time it takes to progress from MCI to AD. This joint modeling provides a more complete understanding of how drugs influence both disease risk and disease progression. A central feature of the framework is weighting mechanism, α, which automatically balances treated and untreated groups as in IPTW, while also enhancing the model’s ability to make accurate ITE estimation. Beyond accurate estimation, CauReL introduces two key advances that move causal modeling from prediction toward interpretation. First, counterfactual-level interpretability allows the model to explain why an individual benefits from a given treatment rather than simply quantifying how much. By generating counterfactual pairs, the predicted outcomes for the same patient under treated and untreated scenarios, CauReL exposes the underlying drivers of treatment response, revealing how factors such as age, comorbidities, genetic variants, or neuroimaging markers shape individual benefit. Second, a white-box ITE estimator transforms these complex counterfactual signals into transparent decision structures. By coupling deep representation learning with uplift forests, CauReL converts high-dimensional model outputs into human-readable subgroup rules. Together, these innovations create a scalable and efficient solution for drug repurposing using real-world data.

Applying this framework to two large EHR networks, OneFlorida+ and the All of Us Research Program, we systematically screened 186 drugs and identified four candidates with consistent and strong protective associations: liraglutide, empagliflozin, entacapone, and amantadine. By focusing on ITEs rather than ATEs, our approach revealed clinically meaningful subgroups that would have been overlooked by traditional population-level methods. Notably, these drugs were associated not only with a lower risk of AD onset but also with a slower rate of conversion from MCI to AD, suggesting that they may extend the period of cognitive stability. Among these, two diabetes-related drugs, liraglutide and empagliflozin, emerged as the top candidates, demonstrating the strongest and most consistent protective effects for AD. Liraglutide, a glucagon-like peptide-1 (GLP-1) receptor agonist widely used to treat type 2 diabetes and obesity^29^, demonstrated the most robust signal across both datasets. Subgroup analyses revealed that its benefits were particularly pronounced in individuals with metabolic comorbidities, such as diabetes and obesity. This pattern is consistent with emerging evidence that metabolic dysfunction and impaired insulin signaling contribute to AD pathogenesis through mechanisms involving neurovascular injury, chronic inflammation, and amyloid accumulation^42–44^. These findings reinforce the rationale for ongoing clinical trials of GLP-1 receptor agonists in neurodegenerative diseases and highlight metabolic pathway modulation as a promising disease-modifying strategy^45–47^. Empagliflozin, a sodium-glucose cotransporter-2 (SGLT2) inhibitor, also emerged as a strong candidate^30–32^. While it is currently prescribed for diabetes and heart failure^48^, its neuroprotective benefits may result from improved vascular health, enhanced glucose regulation, and systemic anti-inflammatory effects^49–51^. Interestingly, its most responsive subgroup included individuals with diabetes, obesity, and cardiovascular comorbidities., suggesting that SGLT2 inhibitors could be repurposed to target overlapping vascular and metabolic pathways implicated in AD.

The two neuroactive drugs, entacapone and amantadine, emerged as promising repurposing candidates for AD, showing a distinctly different mechanistic profile compared to the metabolic drugs. Entacapone, COMT inhibitor approved for Parkinson’s disease, likely exerts its protective effects through dopaminergic modulation^33,34^, a pathway closely linked to cognitive function and neurodegeneration. This finding suggests that patients with Parkinsonian features or dopaminergic dysregulation could represent an ideal target population for repurposing trials, especially given entacapone’s established safety profile and clinical availability. Amantadine, an NMDA receptor antagonist with antiviral and antiparkinsonian properties, demonstrated strong protective effects in individuals with early cognitive decline and psychiatric comorbidities^35^. Its mechanism may involve reducing glutamate-mediated excitotoxicity^52^, a well-known driver of neuronal loss in AD. Subgroup analyses revealed that neuroactive drugs provided broad and consistent benefits across most patient groups, with more than 95% of individuals showing protective effects in our ITE-based waterfall plots. These consistent effects across diverse demographic and clinical subgroups, including variations in age, sex, and comorbidities, indicate that entacapone and amantadine may be suitable for general MCI populations without requiring extensive biomarker stratification. In contrast, the metabolic drugs (liraglutide and empagliflozin) showed greater heterogeneity, with protective effects concentrated in specific subgroups, such as individuals with diabetes, obesity, or cardiovascular comorbidities. This divergence suggests a two-pronged repurposing strategy: neuroactive drugs could be advanced to broad-based, pragmatic clinical trials to evaluate their potential as general preventive or disease-slowing therapies for AD, while metabolic drugs should be pursued through biomarker-guided precision trials to identify the patients most likely to benefit. Our findings extend prior research by addressing key limitations of current drug repurposing efforts. Historically, hundreds of AD clinical trials targeting amyloid-β, tau, neuroinflammation, and oxidative stress have failed^7,53,54^, yielding either null results or modest benefits accompanied by safety concerns. These repeated disappointments underscore the need to look beyond amyloid- and tau-centric models toward systemic and multi-domain interventions. The strong performance of liraglutide and empagliflozin reinforces the growing recognition of metabolic and vascular dysfunction as central drivers of AD pathogenesis. Our results also align with early-phase trials and observational studies that reported cognitive benefits of GLP-1^55,56^ and SGLT2^57,58^ drugs, but they go further by identifying specific subgroups most likely to respond. Similarly, the protective associations of entacapone^59^ and amantadine^60^ extend the literature on neurotransmitter dysregulation in AD and suggest that dopaminergic and glutamatergic pathways remain underexplored targets for disease modification. Collectively, these results broaden the scope of potential therapeutic strategies of AD and highlight the value of RWD in revealing overlooked opportunities.

While promising, our study has several limitations. Residual confounding from unmeasured factors, such as genetic risk or lifestyle variables not captured in EHRs, cannot be fully excluded. The current analysis focuses on single drug exposures, despite the reality of polypharmacy in older adults with MCI, and does not incorporate dosage, treatment duration, or longitudinal adherence patterns that may shape treatment response.

In addition, although the model identifies statistically coherent subgroups, further work is needed to link these patterns to underlying biological mechanisms and clinically actionable profiles. These limitations open several avenues for future development. Integrating multimodal data types, including genomics, imaging, and biomarkers, could strengthen confounding control and support mechanistic interpretation. Extending CauReL to model multi drug regimens and drug drug interactions would better reflect real world prescribing and may identify synergistic combinations. Incorporating dose response structure, treatment timing, and adherence trajectories could refine patient specific predictions and identify optimal therapeutic windows. Finally, embedding CauReL within prospective target trial designs or pragmatic clinical trials will be essential to validate prioritized drugs and to assess their effectiveness in enriched subgroups identified by the model.

## METHODS

### Eligibility criteria

We identified patients with at least one diagnosis of MCI recorded between January 2012 and April 2020 in the OneFlorida+ Clinical Research Network and between January 2009 and June 2020 in the All of Us Research Program. The study protocol was approved by the Institutional Review Board of the University of Florida (IRB202400920) and All of US. To ensure reliable estimation of ITEs, stringent eligibility criteria were applied to define both treatment and comparison groups. All participants were required to be 50 years or older at the time of their first MCI diagnosis and to have at least one year of continuous follow-up, during which they completed three or more clinical visits. Patients with a documented diagnosis of AD or any other form of dementia prior to their initial MCI diagnosis were excluded to ensure accurate disease staging.

### Outcomes

Two clinically relevant outcomes were evaluated. The primary outcome was AD onset, defined as the first recorded diagnosis of AD after the initial MCI diagnosis. The secondary outcome was the time to AD conversion, measured as the number of months between the initial MCI diagnosis and the subsequent AD diagnosis. This dual-outcome design allowed simultaneous evaluation of a drug’s potential to reduce the risk of developing AD and delay disease progression.

### Model Architecture

We developed CauReL to produce transparent, clinically interpretable individual treatment effect (ITE) estimates from EHR data. The model has two components: a representation network that learns balanced patient embeddings and a dual prediction network that estimates potential outcomes under treatment and under control. For patient i∈{1,…,N}, let the baseline covariates be $x_{i}\in R^{d}$, the treatment indicator be ${\text{t}}_{\text{i}}\in \{0,1\}$ (where ${\text{t}}_{\text{i}}=1$ means exposure to the candidate drug after MCI diagnosis and ${\text{t}}_{\text{i}}=0$ means no such drug between MCI and AD onset, and the observed binary outcome be $y_{i}^{\text{inc}}\in \{0,1\}$, with ${\text{y}}_{\text{i}1}=1$ indicating AD onset during follow-up. A secondary continuous outcome $y_{i}^{\text{time}}\in R_{\geq 0}$ counting months from MCI to AD when available.

Under the potential outcome framework (Rubin), each endpoint has two counterfactuals:

$$Y_{i}^{inc}(1),Y_{i}^{inc}(0)\;\text{and}\;Y_{i}^{\text{time}}(1),Y_{i}^{\text{time}}(0),$$

Only the factual pair is observed

$$y_{i}^{\text{inc}}=Y_{i}^{\text{inc}}\left(t_{i}\right),y_{i}^{\text{time}}=Y_{i}^{\text{time}}\left(t_{i}\right).$$

while the complementary values $Y_{i}^{\text{inc}}\left(1-t_{i}\right)$ and $Y_{i}^{\text{time}}\left(1-t_{i}\right)$ are counterfactual. Model predictions are denoted with hats, for example, ${\hat{Y}}_{i}^{\text{inc}}(1)$.

### Representation network

An encoder Φ(⋅), parameterized by $\theta_{\Phi }$, maps baseline covariates $x_{i}$ into a latent embedding $z_{i}=\Phi \left(x_{i}\right)$ via an MLP with batch normalization, ReLU, and dropout. To mitigate confounding, we align the latent distributions of treated and control groups with an IPM penalty:

$$L_{\text{IPM}}={\text{IPM}}_{G}\left(\left\{z_{i}|t_{i}=1\right\},\left\{z_{i}|t_{i}=0\right\}\right),$$

using linear MMD, RBF-MMD, or 1-Wasserstein distance.

### Dual prediction network

From latent representation $z_{i}$, four heads output potential-outcome predictions, one per endpoint and condition:

$${\overset{ˆ}{Y}}_{i}^{inc}(1)=\sigma \left(h_{\text{inc}}^{\left(1\right)}\left(z_{i}\right)\right),{\overset{ˆ}{Y}}_{i}^{inc}(0)=\sigma \left(h_{\text{inc}}^{\left(0\right)}\left(z_{i}\right)\right)$$

$${\overset{ˆ}{Y}}_{i}^{\text{time}}(1)=h_{\text{time}}^{\left(1\right)}\left(z_{i}\right),{\overset{ˆ}{Y}}_{i}^{inc}(0)=h_{\text{time}}^{\left(0\right)}\left(z_{i}\right)$$

Each head is a two-layer MLP with its own parameters. During training, only the condition matching $t_{i}$ contributes to the prediction loss for sample i. At inference, both conditions are evaluated for every patient to obtain the full counterfactual set $\left\{{\hat{Y}}_{i}^{\text{inc}}(1),{\hat{Y}}_{i}^{\text{inc}}(0),{\hat{Y}}_{i}^{\text{time}}(1),{\hat{Y}}_{i}^{\text{inc}}(0)\right\}$.

### Training objective

For each patient, let the factual predictions be

$${\hat{Y}}_{i}^{inc}(1),{\hat{Y}}_{i}^{inc}(0)\in [0,1],$$

$${\hat{Y}}_{i}^{\text{time}}(1),{\hat{Y}}_{i}^{\text{time}}(0)\in R_{\geq 0}$$

Only the condition that matches the observed treatment $t_{i}\in [0,1]$ contributes to the sample’s prediction:

$${\overset{ˆ}{Y}}_{i}^{inc}\left(t_{i}\right)=t_{i}{\overset{ˆ}{Y}}_{i}^{inc}(1)+\left(1-t_{i}\right){\overset{ˆ}{Y}}_{i}^{inc}(0),$$

$${\overset{ˆ}{Y}}_{i}^{\text{time}}\left(t_{i}\right)=t_{i}{\overset{ˆ}{Y}}_{i}^{\text{time}}(1)+\left(1-t_{i}\right){\overset{ˆ}{Y}}_{i}^{\text{time}}(0).$$

The binary prediction loss is the binary cross-entropy between the predicted and observed AD outcomes:

$$L_{\text{inc}}=-\frac{1}{N}\sum_{i=1}^{N}\left[y_{i}^{inc}\;\text{log}\;{\overset{ˆ}{Y}}_{i}^{inc}\left(t_{i}\right)+\left(1-y_{i}^{inc}\right)\text{log}\left(1-{\overset{ˆ}{Y}}_{i}^{inc}\left(t_{i}\right)\right)\right].$$

Let $\Omega_{\text{time}}$ be the set of patients with an observed months-to-conversion label $y_{i}^{\text{time}}\in R_{\geq 0}$. Use mean squared error

$$L_{\text{time}}=\frac{1}{\left|\Omega_{\text{time}}\right|}\sum_{i\in \Omega_{\text{time}}}{\left({\overset{ˆ}{Y}}_{i}^{\text{time}}\left(t_{i}\right)-y_{i}^{\text{time}}\right)}^{2}.$$

The combined multi-task prediction objective is:

$$L_{\text{pred}}=\lambda_{inc}L_{\text{inc}}+\lambda_{\text{time}}L_{\text{time}}$$

where $\lambda_{1}$ and $\lambda_{2}$ control the weighting between binary and continuous outcomes. We first rescale the incidence and time losses to comparable magnitude using running means on the training split. We then choose ($\lambda_{\text{inc}},\lambda_{\text{time}}$) on a small validation grid to maximize a composite objective that rewards incidence discrimination and calibration, minimizes time RMSE, and penalizes representation imbalance.

To balance predictive accuracy with representation alignment, the total loss function is:

$$L_{\text{total}}=L_{\text{pred}}+\alpha L_{\text{IPM}},$$

where α>0 regulates the trade-off between the two objectives. The optimal α is selected by validation, scanning a small grid {0.1,0.5,1.0,2.0,10.0} and choosing the value minimizing validation $L_{\text{total}}$. Model parameters are optimized using the Adam optimizer (learning rate 2 × 10^−3^, weight decay 10^−4^, batch size 128). Gradients are clipped to a maximum L2 norm of 1.0 to ensure stability.

### Individualized treatment effects

The individualized treatment effect for the binary endpoint is defined as

$${\overset{ˆ}{\tau }}_{i}^{inc}={\overset{ˆ}{Y}}_{i}^{inc}\left(1\right)-{\overset{ˆ}{Y}}_{i}^{inc}\left(0\right),$$

which is the model estimated change in AD risk due to treatment for patient i. Negative values indicate benefit; positive values indicate harm. The continuous endpoint ITE is

$${\overset{ˆ}{\tau }}_{i}^{\text{time}}={\overset{ˆ}{Y}}_{i}^{\text{time}}(1)-{\overset{ˆ}{Y}}_{i}^{\text{time}}(0),$$

which is the model estimated change in months to AD after MCI. Positive values here mean longer time to conversion under treatment, that is, treatment delays progression. Population summaries report the average treatment effect, ${\text{ATE}}_{k}=\frac{1}{N}\sum_{i}{\overset{ˆ}{\tau }}_{i}^{k}$ for k∈{inc,time}.

#### Counterfactual directional feature effects

We intervene on one binary covariate at a time, set it to present versus absent while holding all others fixed, recomputed ${\overset{ˆ}{Y}}_{i}^{\text{inc}}(1)$, and average the contrast:

$$DE_{j}^{\text{risk}}=\frac{1}{N}\sum_{i=1}^{N}\left({\hat{Y}}_{i}^{inc}(1)\left|x_{ij}=1-{\hat{Y}}_{i}^{inc}(1)\right|x_{ij}=0\right)$$

Positive values indicate that feature presence raises treated risk, negative values indicate a protective association under treatment. This procedure is counterfactual, not correlational, because it changes the feature value and recomputes the treated outcome with all other covariates held constant.

### CFR-based uplift trees

We translate model predicted ITEs for AD incidence into interpretable subgroup rules using uplift trees. The tree recursively partitions patients by baseline covariates to expose heterogeneity in benefit. At a node with sample set S and size n, a candidate split on covariate j at threshold c produces children $L=\left\{i\in S|x_{ij}\leq c\right\}$ and $R=\left\{i\in S|x_{ij}>c\right\}$. Binary covariates split on present versus absent, and continuous covariates use candidate thresholds at empirical quantiles.

We evaluate (j,c) using a heterogeneity gain that rewards within node homogeneity and between node separation,

$$\text{Gain}\left(j,c\right)=\text{Var}\left({\overset{ˆ}{\tau }}_{S}\right)-\left(\frac{\left|L\right|}{n}\text{Var}\left({\overset{ˆ}{\tau }}_{L}\right)-\frac{\left|R\right|}{n}\text{Var}\left({\overset{ˆ}{\tau }}_{R}\right)\right)+\lambda \left|\mu \left({\overset{ˆ}{\tau }}_{L}\right)-\mu \left({\overset{ˆ}{\tau }}_{R}\right)\right|,$$

where Var(⋅) and μ(⋅) are the sample mean and variance of ITEs and controls the weight on mean separation. We stop when the node size falls below a preset minimum, the depth exceeds a preset limit, or the best gain is nonpositive. The final tree provides human readable paths with leaf level summaries, including mean ITE, confidence interval, and support, yielding transparent subgroup definitions for who benefits and under what baseline conditions.

## Supplementary Files

This is a list of supplementary files associated with this preprint. Click to download.

- SFig.1.jpg
- SFig.2.jpg
- SFig.3.jpg

Supplementary Figure 1. Feature importance in All of Us

**(a)**visualizes subgroup-level heterogeneity stratified by race and sex. The x-axis represents the probability that individuals within a subgroup experience a beneficial treatment effect, while the y-axis represents the probability of high variability or inconsistent effects within that subgroup. Subgroups in the lower-right quadrant exhibit both high benefit and low variability, indicating the most reliable and clinically promising populations.

**(b)**shows ATEs stratified by comorbidity status, including diabetes, hypertension, heart failure, obesity, depression, and stroke. The x-axis lists comorbidities, while the y-axis shows the ATE, with darker bars indicating patients with the comorbidity and lighter bars indicating patients without it. Negative values represent protective associations.

Supplementary Figure 2. Subgroup analyses in All of Us

**(a)**depicts the mean ATEs by age group, where the x-axis represents age categories (50–59, 60–69, 70–79, ≥80), and the y-axis represents the mean ATE. Error bars indicate variability within each age group.

**(b)**shows directional feature importance, where the x-axis lists baseline features and the y-axis shows their directional impact on predicted AD risk. Features with bars pointing to the left are associated with enhanced drug protection, while those pointing to the right are linked to attenuated drug effects.

Supplementary Figure 3. Time of AD conversion in All of Us

**(a)**presents Kaplan-Meier survival curves comparing treated patients and matched controls for time to conversion from MCI to AD. The x-axis represents months since MCI diagnosis, and the y-axis represents the probability of remaining free from AD. Red curves represent treated patients, and blue curves represent matched controls. Clear separation between curves indicates slower progression among treated patients, with all log-rank tests yielding p < 0.05.

**(b)**shows the counterfactual model predictions for each drug. Violin plots depict the predicted distribution of conversion times under two scenarios: no treatment (blue, left) and treatment (red, right). Wider sections indicate higher densities of patients at a given predicted conversion time, and vertical black bars mark the median prediction.

## Data Availability

The datasets generated and/or analyzed during the current study are not publicly available due to institutional restrictions and patient confidentiality but may be available from the corresponding author on reasonable request.

## Code Availability

The CauReL implementation is available as a standalone, Python-based open-source package at https://github.com/QSong-github/CauReL. A companion web server is available at https://caurel.site/ for direct execution.
